# *MET* exon 14 skipping mutations and gene amplification in a Taiwanese lung cancer population

**DOI:** 10.1371/journal.pone.0220670

**Published:** 2019-08-01

**Authors:** Jrhau Lung, Ming-Szu Hung, Yu-Ching Lin, Kam-Fai Lee, Yuan Yuan Jiang, Shao-Lan Huang, Yu-Hung Fang, Ming-Shian Lu, Chin-Kuo Lin, Tsung-Ming Yang, Paul Yann Lin, Meng-Jer Hsieh, Ying Huang Tsai

**Affiliations:** 1 Department of Medical Research and Development, Chang Gung Memorial Hospital, Chiayi branch, Taiwan; 2 Department of Pulmonary and Critical Care Medicine, Chang Gung Memorial Hospital, Chiayi branch, Taiwan; 3 Department of Medicine, College of Medicine, Chang Gung University, Taoyuan, Taiwan; 4 Department of Respiratory Care, Chang Gung University of Science and Technology, Chiayi Campus, Chiayi, Taiwan; 5 Department of Pathology, Chang Gung Memorial Hospital, Chiayi branch, Taiwan; 6 Department of Surgery, Division of Thoracic and Cardiovascular Surgery, Chang Gung Memorial Hospital, Chiayi branch, Taiwan; 7 Department of Anatomic Pathology, Dalin Tzu Chi Hospital, Buddhist Tzu Chi Medical Foundation, Chiayi, Taiwan; 8 Department of Respiratory Care, College of Medicine, Chang Gung University, Taoyuan, Taiwan; 9 Department of Pulmonary and Critical Care Medicine, Chang Gung Memorial Hospital, Linkou Branch, Taiwan; National Cancer Center, JAPAN

## Abstract

Somatic mutations of *MET* gene are emerging as important driver mutations for lung cancers. To identify the common clinicopathological features of *MET* exon 14 skipping mutations and amplification and clarify whether the two *MET* gene alterations cause protein overexpression were investigated using 196 lung cancer samples of Taiwan through real time-qPCR/sequencing, fluorescence in situ hybridization, and immunohistochemistry. The two MET gene alterations are both present in low frequency, ~1%, in the studied lung cancer population of Taiwan. *MET* exon 14 skipping mutations were identified from two early-stage patients, who were both relatively advanced in age, and did not carry other driver mutations. One was an adenocarcinoma and the other was a rare carcinosarcoma. Three gene amplifications cases were identified. Neither of the two *MET* gene alterations would lead to protein overexpression; hence, direct detection in nucleic acid level would be a preferred and straightforward solution for the identification of skipping mutations. The presence of *MET* exon 14 mutations in minor histological types of lung cancers urge to extend screening scope of this mutation in lung cancer and treatment response evaluation in clinical trials. These would be important next steps for the success of MET target therapy in clinical practice.

## Introduction

Lung cancer is the top leading cause of death in human population worldwide. According to the World Health organization, more than 1.6 million people died from lung cancer in 2015 [1]. Early-stage of lung cancer has no distinct and specific symptoms; hence, most patients are diagnosed at the advanced stage. The survival time of advanced-stage lung cancer patients used to be extremely short, mostly within one year. With the discovery of the driver mutations and their corresponding specific target therapeutic drugs, the situation has been much improved for many lung cancer patients. Since these driver mutations converge into several common signaling pathways, simple anatomical and pathological features cannot make clearly discrimination. As a result, precise and efficient molecular diagnosis becomes the only way before proper treatment can be administrated.

MET is a widely expressed receptor tyrosine kinase involved in various cellular processes, including cell growth, proliferation, survival, migration, and differentiation [2]. To serve in different biological functions, MET delivers signals through several different pathways upon ligand binding, including Ras, PI3A/Akt, STAT3, and NF-κB. [3]. In view of the pivotal roles of MET in cell growth, proliferation and survival, deregulation of MET signaling has been identified in different types of cancers including thyroid [4], lung [5], breast [6], gastric carcinoma [7], liver [8], colon [9], kidney [10], and ovary [11]. Numerous MET targeted agents, including monoclonal antibodies and small molecular inhibitors, are currently in clinical development. Emerging data have shown that drastic responses to MET target therapies and survival benefits could be seen in patients with *MET* gene alterations, including high copy number *MET* gene amplification, Y1003X, and exon 14 skipping mutations [12]. The frequency of *MET* amplification is around 1–11% [13], but could reach to higher level in lung cancers when acquired resistance to EGFR TKI or distal metastasis occurs [14, 15]. MET Y1003X mutations, which abolish the binding domain of the E3 ubiquitin-protein ligase, c-CBL, disrupt degradation, and prolong the activation of MET signaling, are extremely rare and are far below 0.1% in lung cancers [16]. The frequency of *MET* exon 14 skipping mutations is around 1–4% in lung cancers [17]. Both *MET* gene amplification and exon 14 skipping mutations are present in all major histologic types of lung cancers, including adenocarcinoma, squamous, adenosquamous, large cell and small cell [15, 16, 18], but are more frequently found in adenocarcinoma and adenosquamous respectively [15, 16, 19]. MET exon 14 skipping mutations tend to be more enriched in relatively elderly population, and mutually exclusive to other lung cancer driver mutations. Screening patients with these clinical relevant *MET* gene alterations would require extensive works and special techniques, such as fluorescence *in situ* hybridization for gene amplification, and PCR based methods coped with specific primer or probe, or sequencing for mutations. To make identification of these patients more efficiently, correlating the *MET* gene alterations with its protein expression level is always extensively attempted. But whether *MET* exon 14 skipping mutations and gene amplification confer higher MET protein expression, or relate with other specific clinicopathological features are still controversial and require more data to conclude [20–22].

In view of the importance of *MET* mutations in the lung cancer pathogenesis and in guiding diagnosis and treatment, the prevalence and clinicopathological features of the two *MET* alterations are investigated using a lung cancer population of Taiwan in the current study. The strengths and weaknesses of various detection methods for the *MET* exon 14 skipping mutations are also discussed.

## Materials and methods

### Specimen collection

A total of 196 formalin fixed paraffin-embedded lung cancer tissue samples collected from 2006 to 2017 with signed informed consent were requested from the tissue bank and biobank of Chang Gung Memorial Hospital, Chiayi. The study was conducted with approval from the institutional review board of Chang Gung Memorial Hospital (Approval No. 201600631B0). For each case, 1.5-mm cores were sampled from two different tumor parts and one adjacent normal part for tissue microarray construction, and seven tissue microarray (TMA) slides were prepared for the downstream FISH and IHC analysis. The clinicopathological features of these samples are summarized in Table 1.

**Table 1:** Clinicopathological features of lung cancer patients in the study.

Variable	Group	No.(%)
Age	Medium	65
	Range	33–96
Gender	Male	123(62.8)
	Female	73(37.2)
Smoking#	NA	116(59.2)
	Heavy	80(40.8)
Stage	IA	36(18.4)
	IB	75(38.2)
	IIA	18(9.2)
	IIB	15(7.7)
	IIIA	39(19.9)
	IIIB	4(2.0)
	IV	9(4.6)
Histology	Adeno	134(68.4)
	Squamous	32(16.3)
	Adenoseqamous	7(3.6)
	Large cell	7(3.6)
	Sarcomatoid	5(2.5)
	Others	11(5.6)
Total		196(100)

^#^Smoking status. NA: 0 pack-year; Light: 1–20 pack year; Heavy: >20 pack-year.

### Authentic plasmid and qPCR standard preparation for *MET* exon 14 skipping mutation detection

The authentic plasmid containing a 529bp fragment encompassing the entire exon 13 to 14 of *MET* (NM_000245.3), and 111bp fragment within exon 4 to 5 of *ACTB* (NM_001101.3) were amplified using primer pair listed in the S1 Table, and cloned into PCRII-Topo. The *MET* exon 14 skipping authentic plasmid was derived from the *MET* wild-type authentic plasmid by two primers (shown in S1 Table) located at each side of exon 13–15 junction in forward and reverse direction, respectively, using a long accurate PCR to amplify the entire *MET* wild type authentic plasmid except exon 14 and self-ligation. These authentic plasmids were all verified by sequencing. The copy numbers of plasmids were estimated according to the molecular weight, concentration of plasmids. The *MET* exon 14 skipping plasmid was serially diluted in a solution of Tris 10 mM, EDTA 1 mM pH = 8, containing 20 ng/ml of yeast total RNA (Thermo Fisher Scientific, Waltham, MA) and 10^5^ copies/μl of each *MET* wild-type and *ACTB* plasmids. Five consecutive dilutions (10^5^, 10^4^, 10^3^, 10^2^ and 10^1^ copies/μl) of *MET* exon 14 skipping mutation plasmids were prepared and defined as 100%, 10%, 1%, 0.1%, and 0.01% respectively.

### Real-time quantitative PCR

**Fig 1:**
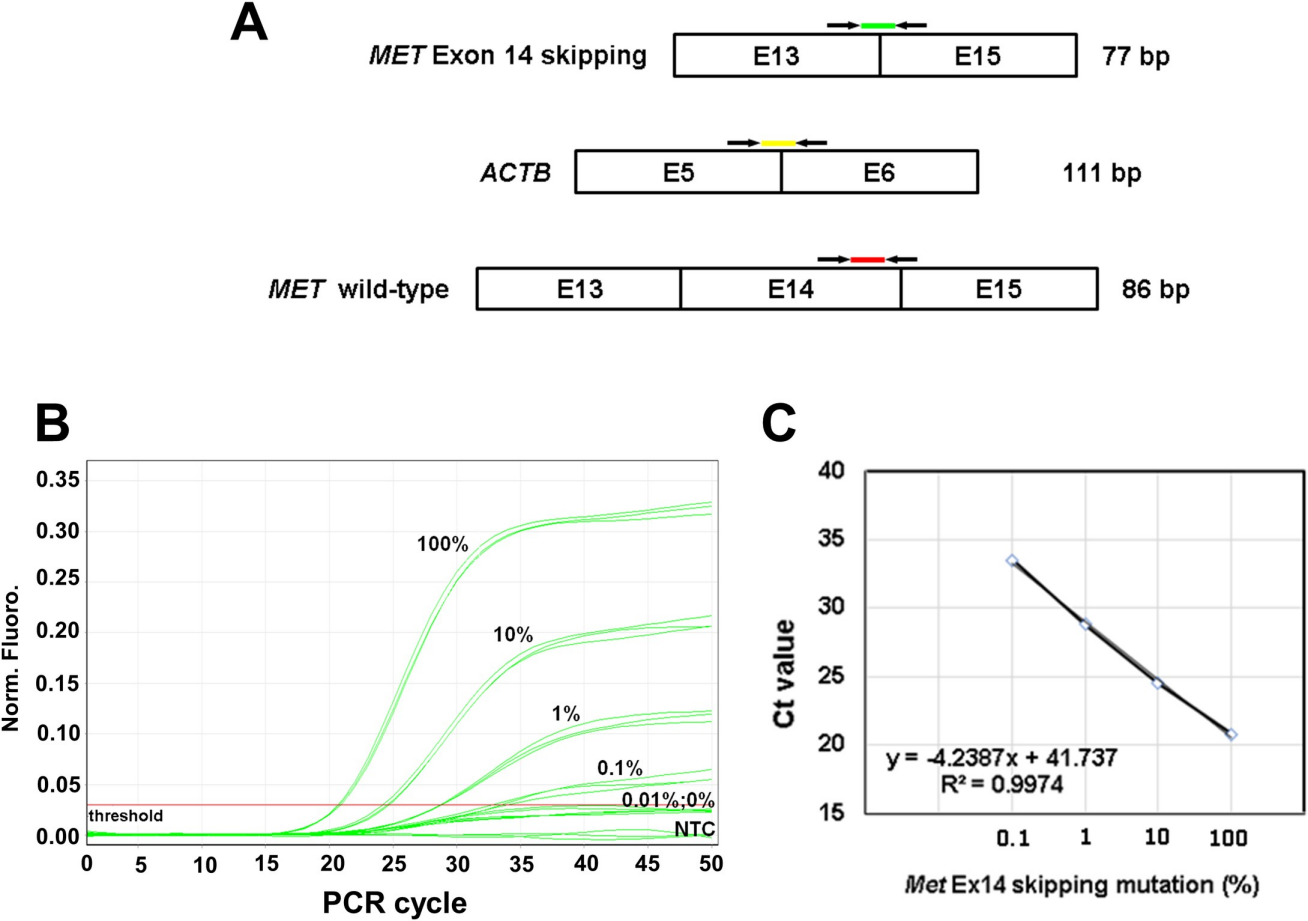
The design and performance of *MET* exon 14 skipping mutation qPCR detection method. (A) The schematic diagram of the primer and probe locations and amplicon sizes used in the qPCR assay. (B) The reprehensive amplification plot of the authentic plasmid standards, (C) The calibration curve for the *MET* exon 14 skipping mutation qPCR assay.

Genomic DNA and total RNA were purified from the FFPE sample using AllPrep DNA/RNA Mini kit (Qiagen, Hilden, Germany) according to the manufacturer’s protocol. For cDNA preparation, 1μg of total RNA from each sample was reverse transcribed using SuperScript III (Invitrogen, Carlsbad, CA) with random hexamer. The primer and probe sequences used in the *MET* exon 14 skipping mutation qPCR assay are listed in S1 Table, and the corresponding locations of these primers and probes are illustrated in Fig 1A. Two separate PCR reactions were adopted to achieve better detection sensitivity by avoiding the competition between the qPCR amplification of *MET* wild-type and exon 14 skipping mutation transcripts. The reaction mixture contains 1× Qiagen QuantiNova Probe master mix (Qiagen, Hilden, Germany), 100 nM of internal control primers, 200 nM of specific primers and TaqMan probes in a 20-μl reaction volume. Real-time PCR was performed on the Qiagen Rotor-gene Q qPCR machine with the following cycling protocol: 95°C for 5 min, 45 cycles of 95°C for 5 s, 60°C for 10 s. The threshold cycle (Ct) value used for analysis was chosen at the normalized fluorescence intensity of 0.02. The qPCR products were electrophoresed in 4% agarose gel to confirm the results.

### *MET* genomic mutation detection

The genomic mutations contributing to the exon 14 skipping mutations were analyzed by PCR amplification of the entire exon 14 and adjacent upstream and downstream intron regions suing two primer pairs listed in S1 Table and Sanger sequencing.

### Fluorescence in situ hybridization

For the detection of *MET* gene amplification, FISH was performed using the *MET*/CEN-7 SureFISH Probe Mix (Agilent Technologies, Glostrup, Denmark) with the Histology FISH Accessory Kit (Agilent Technologies) reagents. The TMA sections were deparaffinized, rehydrated and heat pretreated at 98°C for 10 min. After cooling to room temperature for 15 min, the slides were washed twice with diluted wash buffer. The sections were then air dried and digested with pepsin for 18 min at room temperature. The pepsin digestion was terminated by washing thrice with diluted buffer and graded ethanol dehydration. To perform FISH hybridization, the fluorescence probes were reconstituted with hybridization buffer, overlaid on the TMA slides, and covered with coverslips. Hybridization reaction was performed using the Dako Hybridizer by first heating up to 90°C for 5 min, followed by gradual cooling down to 37°C and incubated for 14–20 hr. Nonspecific binding was removed by washing thrice with diluted Stringent Wash buffer at 60°C. The sections were dehydrated, mounted in medium with DAPI (Invitrogen), rested at room temperature for 24hr in the dark, and then stored at 4°C. FISH signals were observed and counted using a fluorescence microscope (Axio Scope A1, Zeiss, Oberkochen, Germany), and at least 50 non-overlapping nuclei were analyzed for each sample. The *MET* gene amplification will be interpreted if the *MET* gene copy numbers exceed 5 in more than 15% of counted nuclei [13].

### Immunohistochemistry

For detection of MET protein, the anti-total MET rabbit monoclonal antibody (clone SP44, Ventana Medical Systems, Tucson, AZ) was used. The staining was carried out according to the manufacturer’s protocol on the BenchMark XT platform with the Ventana OptiView detection kit. The result was interpreted by a pathologist blinded to the clinical information of these patients. Only signals of staining in the cytoplasm and membrane are interpreted as positive, and the intensity was scored according to a four-tier systems: 0, no staining; 1+, weak; 2+, moderate; and 3+, strong.

## Results

### Establishment of the real time quantitative PCR for *MET* exon 14 skipping mutations

*MET* transcript is widely expressed in cells of epithelial origin; hence, the measurement the *MET* exon 14 skipping mutations with wild-type transcript in a single qPCR reaction together could face competition from the more abundant wild-type counterpart. For this reason, the measurements of *MET* exon 14 skipping mutations and wild type transcripts were performed in two separate reactions. The primer and probe set for *ACTB* gene detection were embedded within each reaction to ensure the appropriate setup and performance of each qPCR reaction. All PCR amplicons were chosen as short as possible to accommodate the highly fragmented nature of RNA from FFPE samples. The amplification blot and standard curve of the *MET* qPCR assay were generated using plasmid authentic standard and are shown in Fig 1B and 1C, respectively. The sensitivity of the established qPCR detection method for *MET* exon 14 skipping reached 0.1%, with the *MET* wild type transcripts being 1000X more abundant, and thus employed to screen the mutations in the lung cancer patient samples.

### Detection of *MET* E14 skipping mutations in archived lung cancer samples

**Fig 2:**
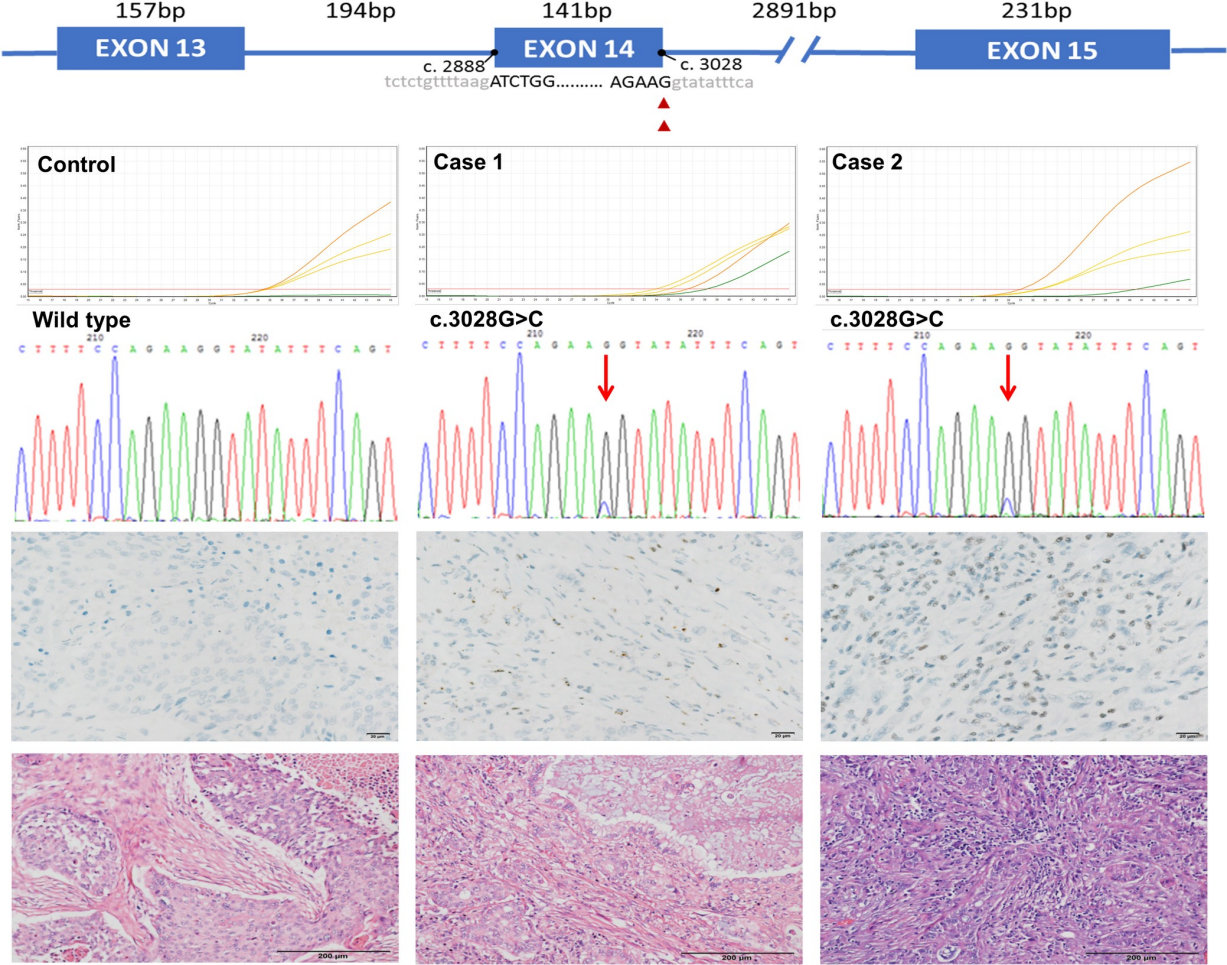
Mutation sites, MET protein expression and histology of two *MET* exon 14 skipping mutation cases. Schematic diagram of the mutation sites of the two *MET* exon 14 skipping mutation cases, qPCR amplification plot, and Sanger sequencing histograms (the first to third rows). The colors of qPCR curves in amplification plots were labeled as the marked colors for each target probe shown in Fig 1. Immunohistochemistry stains (the fourth row, 40X, scale bar = 20 μm); Hematoxylin and Eosin (H&E) stains (the fifth row, 20X, scale bar = 200 μm) for the cases. The case numbers were labeled according to the Table 2.

The qPCR reactions were performed on the 196 collected lung cancer samples and the detection was successful for 170 samples based on the successful amplification of *ACTB* internal control in both qPCR reactions for each sample. The highly fragmented nature of cDNA from FFPE samples would likely compromise qPCR amplification efficiency; hence, the cutoff value for the positivity of the *MET* exon 14 skipping mutations is set to Ct value of 40. Ct values for *ACTB* gene qPCR ranging from 21.68 to 43.4, indicate dramatic variations in RNA qualities of these clinical samples. Among the 170 samples, *MET* wild-type transcripts were successfully detected in 140 samples. Their Ct values ranging from 28.71 to 37.19 indicate comparatively lower *MET* transcript levels than that of *ACTB* in tumor samples. The 30 *ACTB* qPCR positive and *MET* wild-type negative samples were mainly those with late *ACTB* Ct values, which reflect the much less well-preserved *MET* transcripts in these samples. One adenocarcinoma and one carcinosarcoma, a subtype of sarcomatoid carcinoma, were found to express *MET* exon 14 skipping mutation transcripts. The qPCR amplification plots and Hematoxylin and Eosin (H&E) staining images of the two cases are shown in Fig 2.

**Table 2:** The clinicopathological features of patients with *MET* exon 14 skipping mutations, gene amplification and protein overexpression.

Case No	Type	Gender	Age	Stage	Histology	Smoking	Additional mutation
1	Ex14 skipping	F	80	IB	adeno	NA	
2	Ex14 skipping	M	81	IIB	carcinosarcoma	NA	
3	Gene amp	M	50	IIA	large cell	NA	
4	Gene amp	M	78	IB	squamous	+	
5	Gene amp	M	56	IIIA	adeno	NA	EGFR Ex19 del
6	IHC+ (C2+M1+/C1+)*	F	56	IB	adeno	NA	
7	IHC+ (C1+/C1+)	M	51	IA	adeno	+	
8	IHC+ (M1+)	F	51	IB	adeno	NA	EGFR Ex19 del
9	IHC+ (C1+/-)	F	69	IB	adeno	NA	G719S
10	IHC+ (C1+/-)	M	67	IIIA	squamous	+	
11	IHC+ (M1+/-)	F	66	IV	adeno	NA	KRAS (G12C)
12	IHC+ (C1+/-)	M	55	IIIA	adeno	+	
13	IHC+ (C1+/C1+)	M	54	IIIA	adeno	+	L858R
14	IHC+ (C2+/-)	F	40	IB	adeno	NA	L858R
15	IHC+ (C1+/-)	F	67	IIA	adeno	NA	L858R
16	IHC+ (C1+/C1+)	M	75	IB	adeno	+	EGFR Ex20 ins
17	IHC+ (C1+/-)	M	58	IA	adenosqumaous	NA	
18	IHC+ (C1+/C1+)	M	63	IA	adeno	NA	
19	IHC+ (C1 +/-)	F	47	IIIA	adeno	NA	

*IHC positivity was reported according to the subcellular location and intensity of staining for two tumor cores of each sample. C = cytosol staining, M = membrane staining. Intensity was scored according to a four-tier system: 0, no staining; 1+, weak; 2+, moderate; and 3+, strong.

### Identification of genomic mutation confers to exon 14 skipping mutations

Somatic mutations leading to exon14 skipping mutation transcripts in the two qPCR positive samples were further explored by sequencing the entire exon 14 and the adjacent upstream and downstream intron regions using Sanger sequencing. The mutations were also screened within the 30 *MET* wild type qPCR failed and 26 *ACTB* and *MET* qPCR failed samples. The two exon 14 skipping mutation-positive samples were both found to be carrying mutations (c.3802G>C) at the splicing donor site of exon 14. No mutation was identified in the 56 *MET* qPCR failed samples. The prevalence rate of *MET* exon 14 skipping mutations was 1.0% in the studied lung cancer population.

### *MET* amplification

**Fig 3:**
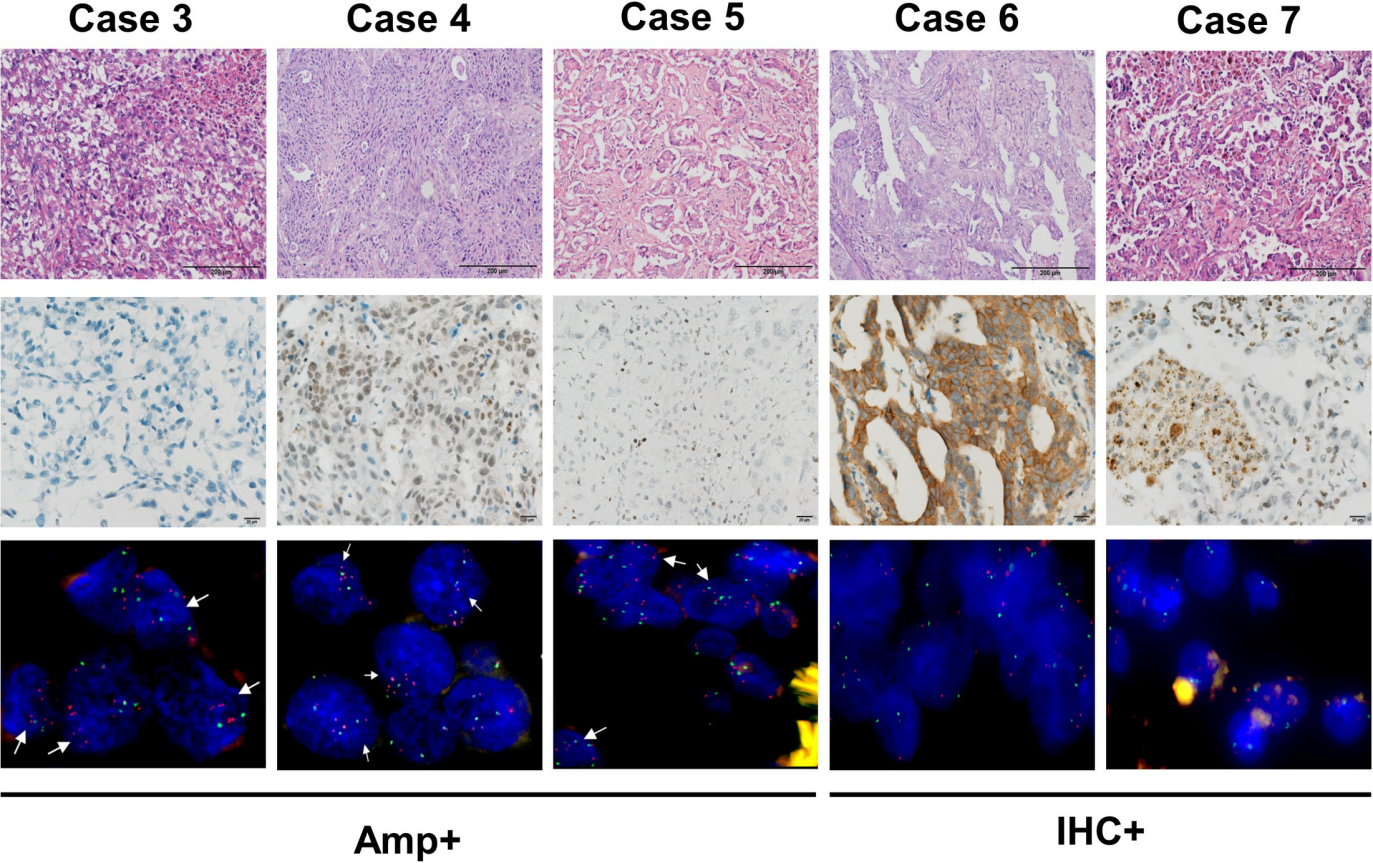
The H&E, Immunohistochemistry and FISH results of two selected MET overexpression cases and 3 *MET* amplification cases. H&E stains (the first row, 20X, scale bar = 200 μm); immunohistochemistry (the second row, 40X, scale bar = 20 μm); FISH (the third row, 63X). The case numbers were labeled according to the Table 2.

*MET* amplification in the 196 lung cancer samples was analyzed using tissue microarray. FISH experiments were successfully performed on all samples. Among them, three were found to have *MET* gene amplification (10 > MET copy number > 5, Fig 3 and Table 2). The levels of amplification for the 3 cases belong to low to intermediate based on the ratio of *MET/CEN7* < 5 [23]. The prevalence rate of *MET* amplification is 1.5% in the studied lung cancer population, and their histology types included adenocarcinoma, squamous, and large cell. The stage IIIA adenocarcinoma case was found to carry an additional EGFR exon 19 deletion mutation (Table 2).

### MET overexpression

The MET protein overexpression was successfully accessed for all 196 samples, and 14 overexpression cases were found (Fig 3 and Table 2). Among them, 12 were from adenocarcinoma, and the other two are squamous and adenosquamous, respectively. The prevalence rates of MET IHC positive cases in adenocarcinoma, squamous and adenosquamous types are 9.1%, 3.0%, and 14.2%, respectively. The staining in ten of IHC positive cases are weak cytoplasmic staining and with heterogeneity, in which 6 samples showed signals only in one tumor cores. For the remaining four positive samples, one showed moderate intensity of cytoplasmic staining in one core, two exhibited weak intensity of membranous staining, and one displayed mixed staining patterns, in which only weak intensity of cytoplasmic staining in one core, and both weak intensity of membranous and moderate intensity of cytoplasmic staining in another. None of the *MET* exon 14 skipping mutations and amplification cases showed protein overexpression.

## Discussion

The current study screened and characterized the clinicopathological features of *MET* exon 14 skipping mutations, gene amplification and protein overexpression using a cohort of resectable lung cancers in Taiwan, and identified two exon 14 skipping mutations (both derived from c3208G>C mutation), three gene amplifications and 14 protein overexpressions. The frequency of the *MET* exon 14 skipping mutations in the current study was around 1%, which is similar to other screening results among East Asian and Caucasian population, ranging from 1 to 4% [16, 19, 20, 24–28]. Although the variations in frequencies could be attributed to the different methodologies used in each study, the variation in composition of patient histology type and stage could also be the causes. Many clinicopathological features of the two *MET* exon 14 skipping mutation cases in this report are similar to those found in previous studies, including, old age (80 and 81), and mutual exclusivity to other driver mutations (*EGFR*, *KRAS*, *ALK*, *ROS1*, *RET*, data not shown) [16, 20, 25]. These findings could suggest that the *MET* exon 14 skipping mutations could preferentially exist in particular patient group. Although *MET* exon 14 skipping mutations have been identified across different major histological subtypes of lung cancers [16], whether this mutation is present in other minority subtypes of lung cancers remains unknown. In the current study, one of the *MET* exon 14 skipping mutation cases identified from a pulmonary carcinosarcoma [29], demonstrated such possibility and highlighted the necessity to broaden the screening scope for this mutation in other minority lung cancer subtypes. Although the clinical efficacy of MET inhibitor for the case is not known, clinical benefit from a rare thoracic histiocytic sarcoma patient receiving crizotinib was reported previously [20]. This merits the inclusion of other *MET* exon 14 skipping mutation cases from other minority histologic subtypes in clinical trials for treatment evaluation.

Like that of *MET* exon 14 skipping mutations, the frequency of the *MET* amplification, 1.5%, in this lung cancer patient group are similar to those reported in other reports, ranging from 2% to 4% [27]. *MET* amplification could also appear in early stage and is independent from other driver mutations; hence, it may support tumorigenesis from early stage and poses oncogenic driver activity. However, crizotinib response only observed in the high *MET* amplification [17], how *MET* amplification in low copies exerts its oncogenic activity and responds poorly to the MET target therapy deserve further elucidation.

Skipping mutations can cause various human diseases [30]. *MET* exon 14 skipping mutations may be one of the few just been uncovered for cancers, because alternative splicing transcripts could be found quite frequently in cancers [31]. Although these skipping mutations could result from various mutations in consensus sequences involved in the splicing event, mutations in the splicing donor site and acceptor site are far more prevalent than those in other sites, and the frequencies of mutations in the donor site exceed those in the acceptor site in more than two folds generally [32]. Currently, at least 165 different mutation types are identified and predicted to be able to generate *MET* exon 14 skipping mutation transcripts. The wide spectrum of skipping mutations could make the molecular diagnosis challenging, since some of these mutations could result in incomplete splicing and several arise from large fragment deletions [16, 33]. These could make RNA-based detection methods more straightforward for screening this mutation. However, the quality of nucleic acids is often seriously deteriorated after the routine formalin-fixed paraffin-embedded tissue processing; hence, there is a significant chance of failure in RNA-based detection of *MET* exon 14 skipping mutations even with a short amplicon design adopted. Using special tissue preservative, such as PAXgene (PreAnalytix, Switzerland) and UMFIX (SakuraFinetek, Torrance, CA), could help solve this situation. With the comprehensive sequencing profiling has become more and more important and affordable for clinical practice, it could be envisaged that adopting data directly from sequencing results using nucleic acids from fresh/frozen cell/tissue samples would be more straightforward for cancer diagnosis and treatment guidance in the future.

Overexpression of MET protein could be frequently observed and is associated with poor prognosis in lung cancer. These observations have led to the impression that MET overexpression triggers hyperactivation of MET signaling and promotes lung cancer tumorigenesis. However, the activation of MET signaling would require more than just overexpression of the receptor alone, which presumably causes the failures of several earlier MET targeted therapy trials performed on patients with MET protein overexpression [34, 35]. Despite the disappointing results, the higher prevalence rate of MET overexpression than *MET* gene alterations in lung cancers frequently observed in studies, including the current one, should prompt further refinement of recruiting criteria, such as including measurement of MET phosphorylation level, in future MET targeted therapy trials using MET protein as a selection marker.

Currently, further accumulation of data is still required before regular use of MET target therapy for lung cancers. This would mainly rely on accurate and efficient identification of suitable patients in clinical trials. The established TaqMan-based qPCR method in the study for detecting *MET* exon 14 skipping mutations could help researchers in efficient identification of the mutations in their works. Although the small sample size is an inherent limitation of the study, it could help in collecting new data at a faster rate and prompt extending our understanding of *MET* alterations in lung cancers, such as whether *MET* exon 14 skipping mutations result in MET protein overexpression or not. Our findings suggest that detection methods for identification of *MET* exon 14 skipping mutations should be selected cautiously in the future, since MET protein overexpression is not always observed in patients with *MET* exon 14 skipping mutation cases; MET protein overexpression is far more prevalent than *MET* exon 14 skipping mutations in lung cancers. For further clarification of the important issue, MET protein expression status in cases involving *MET* exon 14 skipping mutations in various studies are summarized in S2 Table.

In conclusion, *MET* exon 14 skipping mutation and gene amplification are both present in low frequencies, around 1%, in the studied lung cancer population. Due to their low frequencies and do not relate to protein overexpression, detection in nucleic acid level is more suitable for detection of the two *MET* gene alterations. The newly identified *MET* exon 14 skipping mutation from a rare carcinosarcoma case urges extending screening scope for *MET* exon 14 skipping mutations and further evaluation in clinical trials would be an important step for the success of MET target therapy in clinical practice.

## Supporting information

S1 Table Primer and probe sequences used in this study. (DOC)

S2 Table Summary of various *MET* exon 14 skipping mutations studies in lung cancers. (DOC)
