# Supplementary material for: MET exon 14 skipping mutations and gene amplification in a Taiwanese lung cancer population
Source: PLoS One. 2019 Aug 1;14(8):e0220670. doi: 10.1371/journal.pone.0220670 (PMC6675391; doi:10.1371/journal.pone.0220670)
Supplement: S1 Table — (DOC) [file pone.0220670.s001.doc]

**S1 Table. Primer and probe sequences used in this study**

| **Experiment/Targets** | | **Name** | **Sequence** |
| --- | --- | --- | --- |
| **Authentic plasmids**  **construction** | *MET* wild type | Ex13 Forward | 5’-TGGAAGCAAGCAATTTCTTCAAC-3’ |
|  |  | Ex15 Reverse | 5’-CTCTTCCTATGACTTCATTGAAATGC-3’ |
|  | *MET* Ex14 skipping | Ex15 Forward | 5’-ATCAGTCCTAATTCATCTCAGAACG-3’ |
|  |  | Ex13 Reverse | 5’-CTTTAATTTGCTTTCTCTTTTTCAGCC-3’ |
|  | *ACTB* | Ex4 Forward | 5’-ccttccttcctgggcatggagtc-3’ |
|  |  | Ex5 Reverse | 5’-agacagcactgtgttggcgt-3’ |
| **RT-qPCR** | *MET* wild type | Ex14 Forward | 5’- cgaagtgtaagcccaactaca-3’ |
|  |  | Ex15 Reverse | 5’- gaattaggaaactgatcttctgg-3’ |
|  |  | Probe | 5’-ROX-aatggtttcaaatgaatctgtagac-BHQ2-3’ |
|  | *MET* Ex14 skipping | Ex13 Forward | 5’- atgggtttttcctgtggctgaa-3’ |
|  |  | Ex15 Reverse | 5’- gcatgaaccgttctgagatgaa-3’ |
|  |  | Probe | 5’-FAM-ggaaactgatctttaatttgctttctc-BHQ1-3’ |
|  | *ACTB* | Ex4 Forward | 5’-ccttccttcctgggcatggagtc-3’ |
|  |  | Ex5 Reverse | 5’-agacagcactgtgttggcgt-3’ |
|  |  | Probe | 5’-HEX-tgacgtggacatccgcaaagac-BHQ1-3’ |
| **Somatic mut. screening** | *MET*  Intron 13 | Ex13 Forward | 5’-CTACTTGGGTTTTTCCTGTGGCTGA-3’ |
|  |  | Ex14 Reverse | 5’-CTTACAAGCCTATCCAAATGAGGA-3’ |
|  | *MET*  exon 14 | Int13 Forward | 5’-CCCATGATAGCCGTCTTTAACAAG-3’ |
|  |  | Int14 Reverse | 5’-TGGCAGAGGTAAATACTTCCTTTAGGT-3’ |
