# Supplementary material for: MET exon 14 skipping mutations and gene amplification in a Taiwanese lung cancer population
Source: PLoS One. 2019 Aug 1;14(8):e0220670. doi: 10.1371/journal.pone.0220670 (PMC6675391; doi:10.1371/journal.pone.0220670)
Supplement: S2 Table — (DOC) [file pone.0220670.s002.doc]

**S2 Table. Summary of various *MET* exon 14 skipping mutations studies in lung cancers.**

| **Study** | Okuda et al. 2008 [1]. | Awad et al. 2016 [2] | Zheng et al.2016 [3]. | Liu et al. 2016 [4] | Schrock et al. 2016 [5] | Gow et al. 2016 [6] | Lee et al. 2017 [7] | Mignard et al. 2018 [8] | Lambrose et al. 2019 [9]. | Lung et al.  [this study] |
| --- | --- | --- | --- | --- | --- | --- | --- | --- | --- | --- |
| **Histology** | NSCLC | NSCLC (nonsquamous) | NSCLC | NSCLC | Lung cancers | Lung cancers | Adeno | Sarcomatoid | NSCLC | Lung cancers |
| **Detection method** | Sanger sequencing | NGS | RT-PCR/  Sanger sequencing | NGS/  Sanger sequencing | NGS | RT-PCR | RT-PCR/  Sanger sequencing | HRM/  MassArray/NGS | NGS | RT-qPCR/  Sanger sequencing |
| **Frequency** | 1.7%  (3/178) | 3.0%  (28/933) | 1.3%  (23/1770) | 0.9%  (12/1296) | 2.7%  (298/11205) | 3.2%  (27/850) | 2.1%  (17/795) | 6%  (5/81) | 1.4%  (21/1484) | 1.0%  (2/196) |
| **IHC positivity in MET exon 14 skipping mutations** | 1/2 | 25/25 | 19/23 | 3/6 | Not test | 27/27 | 15/15 | 1/5 | Data not shown, but suggests that IHC predicts poorly to *MET* ex14 skipping mutations. | 0/2 |

1. Okuda K, Sasaki H, Yukiue H, Yano M, Fujii Y. Met gene copy number predicts the prognosis for completely resected non-small cell lung cancer. Cancer Sci. 2008;99(11):2280-5. doi: 10.1111/j.1349-7006.2008.00916.x. PubMed PMID: 19037978.

2. Awad MM, Oxnard GR, Jackman DM, Savukoski DO, Hall D, Shivdasani P, et al. MET Exon 14 Mutations in Non-Small-Cell Lung Cancer Are Associated With Advanced Age and Stage-Dependent MET Genomic Amplification and c-Met Overexpression. J Clin Oncol. 2016;34(7):721-30. doi: 10.1200/JCO.2015.63.4600. PubMed PMID: 26729443.

3. Zheng D, Wang R, Ye T, Yu S, Hu H, Shen X, et al. MET exon 14 skipping defines a unique molecular class of non-small cell lung cancer. Oncotarget. 2016;7(27):41691-702. doi: 10.18632/oncotarget.9541. PubMed PMID: 27223439; PubMed Central PMCID: PMCPMC5173088.

4. Liu SY, Gou LY, Li AN, Lou NN, Gao HF, Su J, et al. The Unique Characteristics of MET Exon 14 Mutation in Chinese Patients with NSCLC. J Thorac Oncol. 2016;11(9):1503-10. doi: 10.1016/j.jtho.2016.05.016. PubMed PMID: 27257131.

5. Schrock AB, Frampton GM, Suh J, Chalmers ZR, Rosenzweig M, Erlich RL, et al. Characterization of 298 Patients with Lung Cancer Harboring MET Exon 14 Skipping Alterations. J Thorac Oncol. 2016;11(9):1493-502. doi: 10.1016/j.jtho.2016.06.004. PubMed PMID: 27343443.

6. Gow CH, Hsieh MS, Wu SG, Shih JY. A comprehensive analysis of clinical outcomes in lung cancer patients harboring a MET exon 14 skipping mutation compared to other driver mutations in an East Asian population. Lung Cancer. 2017;103:82-9. doi: 10.1016/j.lungcan.2016.12.001. PubMed PMID: 28024701.

7. Lee GD, Lee SE, Oh DY, Yu DB, Jeong HM, Kim J, et al. MET Exon 14 Skipping Mutations in Lung Adenocarcinoma: Clinicopathologic Implications and Prognostic Values. J Thorac Oncol. 2017;12(8):1233-46. doi: 10.1016/j.jtho.2017.04.031. PubMed PMID: 28502721.

8. Mignard X, Ruppert AM, Antoine M, Vasseur J, Girard N, Mazieres J, et al. c-MET Overexpression as a Poor Predictor of MET Amplifications or Exon 14 Mutations in Lung Sarcomatoid Carcinomas. J Thorac Oncol. 2018;13(12):1962-7. doi: 10.1016/j.jtho.2018.08.008. PubMed PMID: 30149144.

9. Lambros L, Uguen A. MET Immunohistochemistry Should Be Avoided in Selecting Non-small-cell Lung Cancers Requiring MET Exon 14 Skipping Mutation Analysis. Clin Lung Cancer. 2019;20(3):e418-e20. doi: 10.1016/j.cllc.2018.12.002. PubMed PMID: 30606657.
